# Gender Differences in Polychronicity

**DOI:** 10.3389/fpsyg.2019.00597

**Published:** 2019-03-20

**Authors:** André J. Szameitat, Moska Hayati

**Affiliations:** Centre for Cognitive Neuroscience (CCN), Division of Psychology, Department of Life Sciences, Brunel University London, Uxbridge, United Kingdom

**Keywords:** polychronicity, multitasking, individual differences, gender difference, stereotype (psychology)

## Abstract

Polychronicity refers to a personal preference to engage in multitasking. In the current study, we investigated whether male and female participants differed in polychronicity. For this, 167 participants filled out an online questionnaire assessing polychronicity in a variety of ways, including the Multitasking Preference Inventory (MPI). Results showed that women were consistently more polychronic than men. We also found that women showed higher self-rated multitasking abilities, reported to spend more time multitasking, and considered multitasking to be more important in everyday life than men. We conclude that in our sample, which mainly consisted of University students in the United Kingdom, polychronicity shows a significant gender difference.

## Introduction

Polychronicity refers to the preference for multitasking, i.e., to perform one or more tasks concurrently, in contrast to performing only one single task at a time (Poposki and Oswald, 2010). We employ an individualistic definition focussed solely on the individual preference of a person for multitasking, while explicitly leaving out cultural aspects, such as more general time-management, punctuality, or expectations about others’ preferences for multitasking. It is important to note that polychronicity refers to a personal preference, and not to actual multitasking behavior such as multitasking abilities or the amount of time spent multitasking per day (König and Waller, 2010).

Several reasons led to a considerable interest in understanding polychronicity in more detail. For instance, polychronicity is often considered to be a trait, and thus can be seen as a rather stable characteristic to describe personality (Slocombe and Bluedorn, 1999). Furthermore, it has been proposed that virtually every job nowadays requires some form of multitasking, leading to an interest in polychronicity from organizational and managerial perspectives (Bühner et al., 2006; Szameitat et al., 2015). For instance, it has been shown that polychronicity has a unique contribution in explaining job performance (Kantrowitz et al., 2012) and that it modulates the relationship between actual multitasking abilities and job performance (Sanderson et al., 2013).

Due to this relevance of polychronicity, a proper understanding of its determinants is beneficial. A number of studies aimed at identifying variables which may affect polychronicity, such as culture (Adams and van Eerde, 2010; König and Waller, 2010), personality traits (Bhattacharyya et al., 2015), or work-family interface (Korabik et al., 2016). However, we are not aware of any study which has focussed on testing for gender as a factor, i.e., the question whether men and women differ in polychronicity. While gender is occasionally reported as a variable, we are not aware of a study which actually found gender differences with respect to polychronicity.

This finding is somewhat surprising, because there is a strong and widespread belief in the general public that women are better than men at multitasking (Strobach and Woszidlo, 2015; Szameitat et al., 2015). In a previous study of us (Szameitat et al., 2015), we observed some initial evidence (unpublished data) that women might show higher polychronicity than men. Therefore, the aim of the current study was to follow up this initial observation and to test whether there is a gender difference in polychronicity, i.e., the preference to multitask.

The variety of previous studies which did not observe gender effects in polychronicity in terms of participant samples and methodology lets it seem futile to engage in a discussion for potential reasons why no gender effects were observed so far. Thus, we decided to base the current online questionnaire study on one of the most recent developments in assessing polychronicity, the Multitasking Preference Inventory (MPI). The MPI is most suited to assess individual-level polychronicity, while previous measures often also assessed cultural-level polychronicity. The MPI focusses rather strongly on a work environment, e.g., by using terms such as “tasks”, “work on projects,” and “assignments.”

To broaden the focus beyond the work environment, we complemented the MPI by a number of self-developed questions targeting everyday activities (termed “Everyday Multitasking Scenarios”; EMS). We would like to point out that we did not intend to develop a new measure for polychronicity. We merely intended to follow up the incidental observation in our previous data that the nature of the items (work focussed vs everyday activity focussed) might affect whether gender difference can be observed. For this, we generated a questionnaire with a similar structure to the MPI but which takes examples not from the work context but more from everyday life, in particular also from the life as a university student (our main sample). When formulating the questions, we made sure that it is about a *preference* for multitasking and not about the activity itself (e.g., by using the phrase “I like to…”).

Furthermore, we added a question which aimed at providing more in-depth information about what drives peoples’ preference for multitasking. Often, people assume (rightly or not) that multitasking saves time, i.e., one would be finished earlier or would get more work done in the same amount of time. Thus, it might be that people score high on polychronicity because they think that multitasking is more efficient although they actually may dislike multitasking. Alternatively, people may actually just enjoy multitasking while being oblivious to whether it is more efficient – or a combination of both. To our knowledge, previous definitions of polychronicity as the preference for multitasking did not define what exactly drives this preference. For instance, the MPI uses both wordings in its items, i.e., whether one *prefers* or *likes* multitasking. To control for this interpretation, we firstly included a question which asks for polychronicity by explicitly instructing participants that multitasking would not save any time. In addition, we included a direct and explicit question, i.e., “How much do you like multitasking?” These two questions should provide a good insight into the preference for multitasking in terms of *liking* or *enjoyment*.

In addition to these questions aiming to assess polychronicity, we assessed further aspects of peoples’ views on multitasking which might be useful in understanding the polychronicity data. In more detail, to test whether there is an explicit stereotype that women would prefer multitasking, we asked “How much do you think women prefer multitasking more than men?” Because polychronicity is often discussed in relation to actual multitasking behavior, we presented two questions about participants’ self-rated multitasking abilities and time spent on it (“How good do you think you are at multitasking?” and “How many hours per day do you think you spend on multitasking?”). Finally, to assess the perceived relevance of multitasking we asked “How important do you think multitasking is in everyday life?”

Taken together, the aim of the current study was to test whether gender effects in polychronicity can be observed.

## Materials and Methods

### Participants

In total, 167 (89 female, i.e., 53%) participants aged between 18 and 58 years (mean 24, s.d. 7 years) took part in this survey after having given informed consent (Table 1). On average, males were 2.6 years older than females (independent-samples *t*-test; *t*(165) = 2.284, *p* < 0.05; Cohen’s *d* = 0.351)^1^.

**Table 1:** Age information (in years) for the 89 females and 78 males (total *N* = 167) taking part in the study.

Age group	*N* (males)	*N* (females)	Total
18–20	5	16	21
21–30	60	64	124
31–40	8	5	13
41–50	1	3	4
>50	4	1	5
*Mean age*	*25.8*	*23.2*	*24*


Participants were debriefed about the purpose of the study after filling in the questionnaire. This study was carried out in accordance with the recommendations of the Department of Life Sciences Ethics committee, College of Health and Life Sciences, Brunel University London, United Kingdom, and the Declaration of Helsinki, with informed consent from all subjects. The protocol was approved by the above named ethics committee. Participants had to tick a checkbox that they agree to take part in the questionnaire before the questionnaire commenced. If they did not check this box, the study would be terminated. Not all questions were answered by all participants (e.g., because they were not applicable) and we analyzed also partially filled out questionnaires, so that the actual sample size may vary between questions. The questionnaire was distributed mainly among University students in the United Kingdom.

### Materials

The online questionnaire consisted of several parts. First, demographic variables such as gender and age were assessed. Second, the 14 questions of the MPI (Poposki and Oswald, 2010), were presented (Table 2). Participants answered the questions using a five point Likert scale [Strongly Agree (coded as 5), Agree (4), Neutral (3), Disagree (2), Strongly Disagree (1)].

**Table 2:** The Multitasking Preference Inventory (Poposki and Oswald, 2010) as used in the present study.

Item	Males	Females
(1) I prefer to work on several projects in a day, rather than completing one project and then switching to another.	2.86	3.04
(2) I would like to work in a job where I was constantly shifting from one task to another, like a receptionist or an air traffic controller.	2.91	3.16
(3) I lose interest in what I am doing if I have to focus on the same task for long periods of time, without thinking about or doing something else.	3.04	3.52
(4) When doing a number of assignments, I like to switch back and forth between them rather than do one at a time.	2.81	2.91
(5) I like to finish one task completely before focusing on anything else. (R)	3.91	3.69
(6) It makes me uncomfortable when I am not able to finish one task completely before focusing on another task. (R)	3.65	3.43
(7) I am much more engaged in what I am doing if I am able to switch between several different tasks.	2.90	2.98
(8) I do not like having to shift my attention between multiple tasks. (R)	3.23	2.94
(9) I would rather switch back and forth between several projects than concentrate my efforts on just one.	2.6	2.71
(10) I would prefer to work in an environment where I can finish one task before starting the next. (R)	3.67	3.50
(11) I do not like when I have to stop in the middle of a task to work on something else. (R)	3.53	3.56
(12) When I have a task to complete, I like to break it up by switching to other tasks intermittently.	2.69	2.99
(13) I have a “one-track” mind. (R)	3.03	2.54
(14) I prefer not to be interrupted when working on a task. (R)	3.83	3.46


*(R) denotes revere-scored items. Males/Females present the average score across male/female participants for the respective items.*

In addition to the MPI, we presented 12 everyday multitasking scenarios (EMS) to assess polychronicity (Table 3). Participants answered using a 5 point Likert scale [Just like me (coded as 5), Like me (4), Neutral (3), Not like me (2), Not at all like me (1)] and had the further option of “Not applicable” (e.g., when the person did not drive; coded as missing value). Please note that these additional questions should be considered as exploratory pilot work to complement the MPI, they were not intended to form a validated scale on their own (which might be done in future work).

**Table 3:** Self-developed everyday multitasking scenarios (EMS) used in the present study.

Item	Males	Females
(1) When I cook, I like to also do other activities simultaneously, for instance watch the TV rather than solely concentrating on cooking	3.32	3.80
(2) When I drive a car, I like to listen to music and chat to others.	3.59	3.83
(3) When I am working on a project for school/work, I do not like to browse on social media alongside, neither do I like to talk to anybody. (R)	3.46	2.85
(4) When I am shopping, I like to chat on the phone with my friends and family at the same time	2.95	3.24
(5) When I am reading the newspaper on the train and if I receive a text message in that moment, I prefer to respond after I am finished with reading. (R)	3.21	2.61
(6) In the mornings, I like to have my breakfast while getting ready at the same time	2.54	2.63
(7) I like singing songs when I am taking a shower	3.04	3.51
(8) When I clean the house I often find myself switching to different activities such as watching YouTube videos or talking on the phone rather than focusing on cleaning only.	2.78	3.44
(9) I do not like to talk when I am eating. (R)	3.29	2.59
(10) When I am revising for exams in more than one subject, I prefer to frequently switch between the subjects, instead of working on one subject for a prolonged time.	2.83	3.29
(11) When I am working at the computer and a new email arrives, I prefer to first finish what I’m doing before looking at the email, instead of interrupting what I’m doing and looking at it immediately. (R)	3.47	2.69
(12) When I am driving and I have to do something more complicated on the phone (e.g., writing a text) or the SatNAV (e.g., entering the address), I prefer to do this during driving instead of first stopping the car somewhere.	2.66	2.06


*(R) denotes revere-scored items. Males/Females present the average score across male/female participants for the respective items.*

Finally, two direct questions aimed to test for potential gender differences in polychronicity in an explicit way. The first question aimed at assessing the influence of the potential belief that multitasking is more efficient, and was: “Imagine you have to do two tasks, each taking approx. 30 min. You could either do them simultaneously, or one after the other. What would you prefer? Would you prefer to first finish one task completely before switching to the other task, or would you prefer to frequently switch between the tasks? (Please assume that in both cases, it takes the same amount of time, i.e., 1 h, to complete both tasks). Indicate on a scale of 1 (simultaneous) to 5 (one after the other), to what extend you would do it simultaneously or one after the other.” The second question simply was “How much do you like multitasking?” and participants were asked to give their answer on a Likert scale ranging from 1 (Not at all) to 5 (Very much).

With respect to the questions aimed at assessing participants’ views on further aspects of multitasking, we presented the following four questions: (1) “How much do you think women prefer multitasking more than men?” with a Likert answering scale ranging from 1 (not at all) to 5 (very much). (2) “How good do you think you are at multitasking?” with a Likert answering scale ranging from 1 (very bad) to 5 (very good). (3) “How many hours per day do you think you spend on multitasking?,” with an answering scale of 0 h (coded as 1), 0–1 h (2), 1–2 h (3), 3–4 h (4), and over 4 h (5). (4) “How important do you think multitasking is in everyday life?” with a Likert answering scale ranging from 1 (very unimportant) to 5 (very important).

All questions were presented using Qualtrics (http://www.qualtrics.com; Qualtrics Inc., Provo, UT, United States). The questionnaire was distributed and advertised via social media, emails, messages, and online forums.

## Results

### Multitasking Preference Inventory (MPI)

Participants rated the 14 items of the MPI on a scale of 1 (indicating low polychronicity) to 5 (indicating high polychronicity). For the analysis we calculated the overall score by summation of the items. Results (Table 2) showed that women (mean score 40.34, s.d. 9.98) scored significantly higher than men (mean 36.96, s.d. 9.75; independent samples *t*-test *t*(165) = 2.20, *p* < 0.05; Cohen’s *d* = 0.342), indicating a higher polychronicity in women than men. In more detail, the males’ score of 36.96 was significantly lower than the MPI mean score of 42.5 (one-sample *t*-test vs 42.5: *t*(77) = 4.562; *p* < 0.001, Cohen’s *d* = 0.568). The females’ score of 40.34 was also lower than the expected mean score of 42.5, but this difference failed to reach statistical significance [*t*(88) = 1.426, *p* = 0.157, Cohen’s *d* = 0.216].

Internal consistency for the MPI was good in our sample (Cronbach’s alpha = 0.875) and comparable to the values reported for the several samples taken during the development of the MPI (Poposki and Oswald, 2010).

Taken together, the MPI showed a very clear gender effect: women scored higher in polychronicity than men.

### Everyday Multitasking Scenarios (EMS)

Participants rated the scenarios on a scale of 1 (indicating low polychronicity) to 5 (indicating high polychronicity). For the analysis we calculated the overall score by averaging of the items. Women (mean 3.29, s.d. 0.56) showed significantly higher average ratings than men (mean 2.90, s.d. 0.68; Table 3; independent samples *t*-test *t*(161) = 4.06, *p* < 0.001; Cohen’s *d* = 0.633). In more detail, the females’ score of 3.29 was significantly higher than the EMS mean score of 3 (one-sample *t*-test vs 3: *t*(85) = 5.222, *p* < 0.001, Cohen’s *d* = 0.518), while the men’s score was numerically below the EMS mean [*t*(76) = 1.806, *p* = 0.075, Cohen’s *d* = 0.147].

Internal consistency for the EMS was acceptable (Cronbach’s alpha = 0.731) (Nunnally, 1978). To calculate Cronbach’s alpha, we excluded all participants who answered “Not applicable” for one or more of the 12 EMS questions, which reduced the sample size from 168 to 113.

Taken together, also the self-developed EMS showed that women scored higher in polychronicity than men.

### Direct Questions

The first question was “Imagine you have to do two tasks, each taking approx. 30 min. You could either do them ^∗^simultaneously^∗^, or one after the other. What would you prefer? […]” with a Likert answer scale ranging from 1 (simultaneous) to 5 (one after the other). Please note that the coding is reversed here, i.e., higher polychronicity is reflected by lower scores. Women (mean 3.35, s.d. 1.34) scored significantly lower than men (mean 3.82, s.d. 1.19; independent samples *t*-test, *t*(149) = 2.25, *p* < 0.05; Cohen’s *d* = 0.369), indicating higher polychronicity in women. In more detail, both males and females showed scores significantly higher than the mean of 3 (one-sample *t*-test vs 3 males: *t*(71) = 5.799, *p* < 0.001, Cohen’s *d* = 0.689; females: *t*(79) = 2.333, *p* < 0.05, Cohen’s *d* = 0.261).

The second question was “How much do you like multitasking?” with a Likert answer scale ranging from 1 (Not at all) to 5 (Very much), i.e., higher scores indicate higher polychronicity. Women (mean 3.24, s.d. 1.11) scored significantly higher than men (mean 2.78, s.d. 1.25; *t*(151) = 2.40, *p* < 0.05; Cohen’s *d* = 0.387), indicating again higher polychronicity in women. In more detail, women showed numerically higher scores than the mean (expressing liking) while men showed numerically lower scores than the mean (expressing disliking), but both effects failed to reach statistical significance (one-sample *t*-tests vs 3; females *t*(80) = 1.902, *p* = 0.061, Cohen’s *d* = 0.216; males: *t*(71) = 1.512, *p* = 0.135, Cohen’s *d* = 0.176).

In addition to the reverse coding of some items in the MPI and EMS, the last two questions also incorporated opposite coding (lower scores reflect lower vs higher polychronicity, respectively), showing that the observed effects are not simply a matter of how the Likert scales are employed by men and women.

Taken together, also if participants are asked explicitly about their preference, women show higher polychronicity.

### Correlations Between Measures of Polychronicity

In total we assessed polychronicity by four different measures, the MPI, EMS, and two direct questions. Because the MPI and EMS are based on summed or averaged scores, respectively, we used a parametric bivariate Pearson’s correlation, while we used non-parametric Spearman’s rho for all correlations with the two direct questions, which were each assessed by a single 5-point Likert scale item. Results in Table 4 show that all measures of polychronicity correlated significantly with each other (all *p* < 0.01), with the only exception of the two direct questions (*p* = 0.170).

**Table 4:** Correlations between the different polychronicity measures.

	EMS	Simultaneous vs Serial	Do you like multitasking?
MPI	*r* = 0.577^∗∗∗^	*rho* = -0.278^∗∗^[1]	*rho* = 0.474^∗∗∗^
EMS	1	*rho* = -0.393^∗∗∗^	*rho* = 0.405^∗∗∗^
Simultaneous vs Serial		1	*rho* = -0.112^n.s.^


*Note that the different signs of the correlations are due to different coding (higher polychronicity reflected by higher values for MPI, EMS, and “Like Multitasking” but by lower values in “Simultaneous vs Serial”. ^∗^*p* < 0.05; ^∗∗^*p* < 0.01; ^∗∗∗^*p* < 0.001; ^*n.s.*^*p* = 0.170. All significant correlations remain significant after Bonferroni correction for 6 tests, except for ^*[1]*^. All *N* between 151 and 163. *r* = Pearson’s bivariate correlation. *rho* = non-parametric Spearman’s rho correlation. All significant correlations remain significant when calculated separately for males and females and (for MPI with EMS) when Spearman’s rho is calculated instead of Pearson’s *r*.*

### Further Aspects of Multitasking

To assess participants’ views on further aspects of multitasking, we presented the following four questions. To test whether there is an explicit stereotype that women would prefer multitasking, we asked “How much do you think women prefer multitasking more than men?” with a Likert answering scale ranging from 1 (not at all) to 5 (very much). Interestingly, both male (mean rating 3.79, s.d. 1.11) and female (mean 3.74, s.d. 0.99) participants stated that they think that women would prefer multitasking (one-sample *t*-tests vs a test value of 3 (middle point of the scale) males *t*(71) = 6.04, *p* < 0.001, Cohen’s *d* = 0.712; females *t*(80) = 6.69, *p* < 0.001, Cohen’s *d* = 0.748). However, male and female ratings did not differ significantly from each other (two-sample *t*-test *t*(151) = 0.30, *p* = 0.766; Cohen’s *d* = 0.048), indicating that the believe that women are more polychronic is the same for both genders.

Next, we presented two questions about their own multitasking behavior. First, we asked “How good do you think you are at multitasking?” with a Likert answering scale ranging from 1 (very bad) to 5 (very good). Male participants rated their own multitasking abilities as average (mean 3.10, s.d. 1.18; one-sample *t*-test vs 3 (middle point of scale) *t*(71) = 0.70, *p* = 0.486, Cohen’s *d* = 0.085), while females rated their abilities as significantly above average (mean 3.52, s.d. 1.05; one-sample *t*-test vs 3 *t*(80) = 4.44, *p* < 0.001, Cohen’s *d* = 0.495). This gender difference, i.e., females assigning themselves higher multitasking abilities than males, was significant [*t*(151) = 2.34, *p* < 0.02; Cohen’s *d* = 0.378]. A similar pattern emerged for the question “How many hours per day do you think you spend on multitasking?,” with an answering scale of 0 h (coded as 1), 0–1 h (2), 1–2 h (3), 3–4 h (4), and over 4 h (5). Females (mean 3.84, s.d. 0.99) reported to spent more time multitasking than males (3.22, s.d. 1.21; two-sample *t*-test *t*(151) = 3.46, *p* < 0.001; Cohen’s *d* = 0.557).

Finally, we asked “How important do you think multitasking is in everyday life?” with a Likert answering scale ranging from 1 (very unimportant) to 5 (very important). Females (mean 3.89, s.d. 0.98) assigned significantly higher importance to multitasking than males (mean 3.49, s.d. 0.93; two-sample *t*-test *t*(151) = 2.58, *p* < 0.05; Cohen’s *d* = 0.419).

To test whether there is a relationship between the above questions and polychronicity, we calculated non-parametric Spearman’s rank correlations (Table 5). Higher polychronicity was associated with higher self-rated multitasking abilities, higher perceived importance of multitasking, and more (self-rated) time spent multitasking.

**Table 5:** Non-parametric Spearman’s rho correlations between the ‘further aspects’ items and the measures of polychronicity.

	MPI	EMS	Simultaneous vs. Serial	Do you like multitasking?
How good do you think you are at multitasking?	*rho* = 0.378^∗∗∗^	*rho* = 0.455^∗∗∗^	*rho* = -0.120^n.s.^	*rho* = 0.569^∗∗∗^
Importance of multitasking for everyday life	*rho* = 0.294^∗∗∗^[1]	*rho* = 0.267^∗∗^[2]	*rho* = -0.016^n.s.^	*rho* = 0.448^∗∗∗^
Time spent multitasking	*rho* = 0.394^∗∗∗^	*rho* = 0.360^∗∗∗^	*rho* = -0.188^∗^[3]	*rho* = 0.365^∗∗∗^.


*^∗^*p* < 0.05; ^∗∗^*p* < 0.01; ^∗∗∗^*p* < 0.001; ^*n.s*^*p* > 0.05. All correlations remain significant when Bonferroni correction for 12 tests is applied, except for [3]. When the correlations are calculated (without Bonferroni correction) separately for males and females, males show the same pattern of significant correlations, except for [3] (*p* = 0.296). Females also show the same pattern, except for [1] (*p* = 0.051), [2] (*p* = 0.157), and [3] (*p* = 0.093). In all instances of non-significance ([1], [2], and [3]), the direction of the correlation is consistent with the overall pattern.*

Raw data can be found in Supplementary Table S1.

## Discussion

In all our measures women showed significantly higher polychronicity than men. In the MPI and EMS all except for one of the 26 items showed the effect numerically, indicating that this finding is highly consistent. The direct questions which explicitly asked for multitasking preference further supported these findings. Finally, our further questions showed that women not only indicated higher polychronicity than men, but also self-rated their own multitasking abilities higher, reported to spend more time at multitasking, and assigned a higher importance to multitasking in everyday life.

The present data clearly showed higher polychronicity in women than in men. While this relative comparison between genders is evident, it might not necessarily imply that women are truly polychronic. In other words, do women really prefer and/or like multitasking, or are they just less disapproving of it than men? This question can be answered when looking at the absolute scores of men and women. All employed scales and questions had answer scales which had two opposite poles (e.g., like vs dislike) with a clearly defined mid-point (e.g., neither like nor dislike). Therefore, it seems reasonable to interpret statistically significant deviations from the mid-point as an explicit preference for either multitasking or single-tasking. Males show a rather clear picture: All scores indicate a preference for single-tasking (with the MPI and the question on Serial vs Simultaneous processing being statistically significant, the other two non-significant, see Results section for details). For women the picture is mixed: In the EMS, females scored significantly above the mean, i.e., indicating an actual preference for multitasking. In the question “I like multitasking” females show higher scores than the mean, again indicating a preference for multitasking, but this failed to reach significance (*p* = 0.061). In the other two measures females, like males, showed scores indicative of single-tasking (with the question about Serial vs Simultaneous processing being statistically significant and the MPI non-significant). Taken together, while men on average prefer single-tasking over multitasking, women seem to be more ambiguous by sometimes showing explicit preferences for multitasking and sometimes for single-tasking.

The above described results are the mean scores across the whole sample. This sample will most likely consist of some people preferring multitasking (i.e., scoring above the mid of the scale), some preferring single-tasking (below mid of the scale) and some being ambiguous about it (being in the middle of the scale). The above means suggest that the relative distribution of the different types of people differs for males and females. This was indeed the case: For the EMS, 59 out of 86 (69%) women indicated a preference for multitasking (mean EMS score > 3), but only 38 out of 77 (49%) of males did. The same was true for the MPI (sum of MPI items > 42.5) with 39% of women and 29% of men preferring multitasking, and the question how much they like multitasking [39% of women like it (i.e., a score of 4 or 5 on the 5-point Likert scale), while only 28% of men do]. For the question of Serial vs Simultaneous task processing with the explicit notion that multitasking would not save time, only 17 out of 86 (20%) women and only 8 out of 77 (10%) males preferred the simultaneous performance (again, scores of 4 or 5).

The above results bear some interesting implications. First, despite all measures aiming to assess polychronicity, the nature of the question or items in a questionnaire may heavily influence the actual preference for multitasking. The EMS contains some items with a rather liberal interpretation of multitasking, for instance listening to music or singing during an activity. Such activities impose low cognitive demands (Szameitat et al., 2015) and therefore, people are more inclined to engage in them (69% of women and 49% of males, see above). The MPI focusses more on the work context and virtually exclusively uses the terms *task* and *project*. They may imply much more cognitively straining activities and people are less likely to prefer them in multitasking (39% of women and 29% of men). Interestingly, the exactly same numbers were observed for the question whether people like multitasking, which may suggest that in this question people considered activities as tested in the MPI, supporting the validity of the MPI (note that all participants first did the MPI, then the EMS, then the question about serial vs simultaneous processing, and only then the question How much do you like multitasking, i.e., it is unlikely that the similar values are due to a simple carry-over effect). Finally, when explicitly pointing out that multitasking would not save any time, preference for it drops to only 20% for women and 10% for men. This might indicate that the preference for multitasking is at least partially driven by thinking that it is more efficient, and not necessarily purely by a liking of the activity. Future research should, therefore, specify polychronicity in more detail and in particular consider the drivers for polychronicity. Another noteworthy characteristic of the above percentages is that although the absolute numbers of polychronic people varies profoundly between 69 and 10%, the gender difference is rather constantly 10–20%, corroborating the conclusion that women show relative higher polychronicity than men and that more women explicitly prefer multitasking than men. While the above interpretations can still be seen preliminary at the present time, they may spark interesting research into the exact underpinnings of polychronicity.

It is important to note that our data are self-rated measures, and that there is little evidence that women actually would engage more in multitasking or that they would be better at it (Szameitat et al., 2015). For instance, Buser and Peter (2011) conducted a study in which participants had to perform several office tasks in different regimes enforcing either single- or multitasking, or leaving the decision to the participants. The authors found that there were no gender differences in multitasking performance or in the propensity to engage in multitasking. This is consistent with other studies showing no profound gender difference in more office like tasks (Paridon and Kaufmann, 2010) and more cognitive psychology experimental paradigms (Redick et al., 2012). Thus, the currently observed correlation between self-rated multitasking abilities and polychronicity should not be taken as an indicator of actual multitasking abilities or job performance.

During the development of the MPI, the authors also included a computerized multitasking paradigm to test for a relation between multitasking abilities and polychronicity as assessed by the MPI (Poposki and Oswald, 2010). They found that MPI scores did not predict performance in the task, which is in agreement with other studies failing to show that polychronicity can predict actual performance (König et al., 2005). However, MPI scores did predict enjoyment of the computerized multitask, which might suggest that measures of polychronicity may be more suitable in predicting job satisfaction than job performance (Poposki and Oswald, 2010).

The present study does not allow inferences on the causality of the relationship. We would just like to note that in the wider sense, there are three important variables: (a) the preference for multitasking, i.e., polychronicity, (b) actual time spent with multitasking, i.e., practice, and (c) how good someone is at multitasking, i.e., abilities. Causal relationships between these variables are conceivable and seem plausible, but as discussed above, polychronicity (a) seems to be neither related to practice (b) nor abilities (c). Further research is required to confirm that there are indeed no associations between these variables or whether better or other measurements are needed to unveil them.

The data on self-rated multitasking behavior is consistent with our previous study (Szameitat et al., 2015). In particular that study also found that women in the United Kingdom rated their own multitasking abilities higher than men, and also reported to spend more time multitasking. An interesting notion is that Szameitat et al. (2015) investigated several countries, and only the United Kingdom and Germany exhibited a gender effect in self-rated multitasking abilities. If one assumes that polychronicity and self-rated multitasking abilities are related to each other (irrespective of the nature of this relationship), this may mean that gender differences in polychronicity may be apparent only in certain countries. However, this needs to be clarified by future research.

To our knowledge, this is the first study to report gender differences in polychronicity. Given the strength of the effects this seems a bit surprising. However, there may be several reasons why gender differences have not been reported before. For instance, most studies do not report gender as a factor, so that gender differences may have been existent in previous research without noticing them. But there are still some studies which reported gender as a factor, and in these studies gender had no significant effect on polychronicity. Besides the great variety in samples, contexts, and methodological approaches already mentioned in the Introduction, we would like to point to two further factors. First, polychronicity may decrease with age (Poposki and Oswald, 2010) so that our comparatively young participant sample might be particularly sensitive to detect differences as compared to samples taken in work places which span the whole working age range. Second, although the United Kingdom, where virtually all our participants came from, is considered to be a monochronic culture, it has been suggested to show more polychronic characteristics than for instance the United States (Van Everdingen and Waarts, 2003), again making our sample a bit more sensitive to detect differences. After all, in the extreme case of a 100% monochromic culture, all polychronicity scores would converge to zero, and gender differences could not exist. However, future research is clearly needed to replicate the current findings in the United Kingdom and to test whether they can be generalized to other countries.

Our main measure of polychronicity was the MPI. We complemented the MPI by the self-developed 12-item EMS plus some further individual questions. We introduced the EMS because incidental observations in our data (unpublished) gave rise to the speculation that gender differences may be evident in the EMS but not the MPI. However, this turned out to be not the case, the MPI also showed a significant gender effect. Therefore, we think that the EMS is not required as a new or additional tool to measure polychronicity. In the current study, it proved valuable to confirm the data of the MPI, but we do not feel a need to follow up the use or development of the EMS.

Taken together, we were able to demonstrate that in our sample, which mainly consisted of University students in the United Kingdom, women have higher polychronicity than men. Polychronicity correlated positively with self-rated multitasking abilities, but we suggest that this correlation possibly does not generalize to actual multitasking abilities.

## Author Contributions

AS and MH developed the study, and analyzed and interpreted the findings. MH collected the data and provided feedback on the manuscript. AS wrote the manuscript. This work is based on MH’s undergraduate final year B.Sc. dissertation.

## Conflict of Interest Statement

The authors declare that the research was conducted in the absence of any commercial or financial relationships that could be construed as a potential conflict of interest.

## Footnotes

1 Please note that it is highly unlikely that this age difference can account for potential gender differences in polychronicity, because age did correlate neither with the MPI nor with the EMS, calculated separately for each gender or as a whole sample (all *p* > 0.160).

## Supplementary Material

Table S1 Raw data of all participants as provided by Qualtrix.
